# Deep Learning Algorithms for the Diagnosis and Staging of Retinopathy of Prematurity: A Systematic Review and Meta-Analysis

**DOI:** 10.7759/cureus.115894

**Published:** 2026-09-07

**Authors:** Anna Nikolaidou, Stella Moutzouri, Konstantinos Benekos, Theodora Gianni, Christos Frantzidis, Eirini Maliagkani, Andreas Symeonidis, Christos Bergeles, Anna-Bettina Haidich, Asimina Mataftsi

**Affiliations:** 1 Institute for Ophthalmic Research, University of Tübingen, Tübingen, DEU; 2 2nd Department of Ophthalmology, School of Medicine, Faculty of Health Sciences, Aristotle University of Thessaloniki, Thessaloniki, GRC; 3 Department of Ophthalmology, University Hospital of Ioannina, Ioannina, GRC; 4 Department of Ophthalmology, "Agia Sophia" Children's Hospital, Athens, GRC; 5 School of Computer Science, University of Lincoln, Lincoln, GBR; 6 1st Department of Ophthalmology, National and Kapodistrian University of Athens, School of Medicine, Athens, GRC; 7 School of Electrical and Computer Engineering, Aristotle University of Thessaloniki, Thessaloniki, GRC; 8 King's College London, School of Biomedical Engineering and Imaging Sciences, London, GBR; 9 Department of Hygiene, Social-Preventive Medicine and Medical Statistics, School of Medicine, Faculty of Health Sciences, Aristotle University of Thessaloniki, Thessaloniki, GRC

**Keywords:** artificial intelligence, deep learning (dl), diagnosis, pediatric ophthalmology, retinopathy of prematurity (rop), staging

## Abstract

The objective of this systematic review and meta-analysis is to evaluate artificial intelligence (AI) algorithms that diagnose and stage retinopathy of prematurity (ROP). Diagnostic accuracy studies reporting metrics of algorithms using fundus images for ROP identification and staging were included and evaluated. Meta-analysis was conducted for ROP identification studies. Fourteen studies were included in the systematic review and ten studies in the meta-analysis. In total, the studies included in the meta-analysis dealt with 37,057 images. AI showed a high performance in detecting ROP with pooled sensitivity equal to 95% (95% CI 91.5%-97.1%), while the respective value concerning specificity was 97.4% (95% CI 95.3%-98.6%) for ROP identification. The heterogeneity index I² showed a moderate level of heterogeneity, equal to 0.75. Most of the studies lacked bias control for participants’ selection to allow clinical setting applicability. Our research demonstrates that AI algorithms have the potential to become a useful tool in accurately diagnosing and grading ROP. ROP screening can be enhanced by the application of AI but requires special attention regarding bias and real-world implementation.

## Introduction and background

The frequency of retinopathy of prematurity (ROP) rises along with the increasing newborn survival rates. Timely management of ROP requires frequent eye examinations by skilled ophthalmologists, which places a burden on disease detection in underserved areas that lack trained professionals [1]. Late detection is thought to be the root cause of vision loss due to ROP [2]. Artificial Intelligence (AI) applications, such as machine learning classifiers and deep learning techniques, hold great promise for accelerating, simplifying, and enhancing the present detection approach of ROP [3], with diagnostic and staging algorithms being in the spotlight of research. Early detection allows for timely intervention and treatment, which can significantly improve the outcomes for affected infants, enhance the quality of life, and reduce years lived with disability associated with ROP-related visual impairments. Secondly, ROP is a progressive condition that can rapidly advance to the most severe stage if not identified and treated in a timely fashion.

AI-based systems can serve as diagnostic tools to identify images that require further assessment from ophthalmologists, improving the accuracy and efficiency of diagnosis. In clinical practice, several ROP-related features are used for detection, including the stages of ROP [4], disease [5], and vascular severity [6]. These features help assess the severity and progression of the disease. Additionally, AI-assisted telemedicine approaches [7] coupled with smartphone-based fundus imaging collection [8] are being considered to improve ROP diagnosis and facilitate follow-up comparisons. However, the implementation of these technological advancements faces challenges due to structural problems within healthcare systems in low- and middle-income regions, which may delay their benefits for these populations.

Detection and staging of ROP are usually paired with high interobserver variability, while diagnosis becomes quantifiable and objective with AI [9]. AI algorithms have the potential to be a time-saving and cost-effective way of accurately diagnosing ROP. Integrating AI has been proposed to improve efficiency and reduce the workload in ROP clinical practice. The objective of this review and meta-analysis is to analyze the diagnostic performance of AI algorithms in ROP identification and staging.

## Review

Methods

The present meta-analysis was registered at the International Prospective Register of Systematic Reviews (PROSPERO) [10] (reference number CRD42023387110). The reviewers evaluated studies according to the Preferred Reporting Items for Systematic Reviews and Meta-Analyses (PRISMA) 2020 Guidelines [11]. This systematic review and meta-analysis included studies published before January 2024 that reported on the accuracy, sensitivity, specificity, and/or area under the receiver operating characteristic (ROC) curve (AUC) of AI-based algorithms for diagnosing and/or staging ROP (diagnostic accuracy studies). Only those that used at least 50 fundus images were eligible. Three online databases were utilized, including Medical Literature Analysis and Retrieval System Online (MEDLINE) (via PubMed), Scopus, and the grey literature via the ProQuest Dissertations & Theses Global database. The full PubMed search string is provided in Appendix 1. Quality assessment of included records was conducted using the Prediction Model Risk of Bias Assessment Tool (PROBAST) [12], a tool for assessing the risk of bias (ROB) and applicability of diagnostic and prognostic prediction model studies by the two reviewers (AN and SM). PROBAST is divided into four domains: participants, predictors, outcome, and analysis. These domains comprise a total of 20 signaling questions to assist organized judgment of ROB. The two reviewers' disagreements were adjudicated by senior researchers.

A thorough data extraction was completed by the two reviewers (AN and SM), including year of publication, sample characteristics, country where the study was conducted, type of algorithm, camera used, databases used, number of fundus images, comparison with experts’ results, limitations reported, and algorithms’ indices: accuracy, sensitivity, specificity, AUC for ROP diagnosis, and staging. Categorical variables were expressed as frequency and relative frequency. Continuous variables were expressed as mean (± standard deviation) for normally distributed variables and median (interquartile range or Q1-Q3) for non-normally distributed variables. All estimations were based on the extraction of the reported counts of true positive (TP), true negative (TN), false positive (FP), and false negative (FN).

The meta-analysis of diagnostic accuracy was based on the bivariate model, and summary statistics were estimated for sensitivity, positive likelihood ratio (LR+), negative likelihood ratio (LR-), and false positive rate (FPR). The meta-analysis of diagnostic and screening test software (Meta-DiSc, Version: 2.0, Universidad Complutense, Barcelona, Spain) [13] was used to make pooled estimates and design the ROC plane and summary receiver operating characteristic (SROC).

We also constructed hierarchical summary receiver operating characteristic curves (HSROC) using Stata version 13.0 (StataCorp LLC, College Station, TX, USA). The LR+ was defined as the probability of a positive outcome given a positive screening. Similarly, the LR-, was defined as the probability of a negative outcome given a negative screening. The cut-off for an acceptable LR- value is less than 0.2. We performed a formal assessment of publication bias through a regression analysis by constructing a Deeks funnel plot [14]. Statistical heterogeneity among studies was reported as follows: I^2^ statistic <50%: low, 50-75%: moderate, >75%: high heterogeneity. Statistical significance was set at 0.05, and all calculations were also performed using Stata version 13.0, Metandi module; StataCorp and R Software, version 4.3.2 (R Foundation for Statistical Computing, Vienna, Austria) [15].

Results

The initial literature search yielded 1,346 articles from two peer-reviewed databases, grey literature, and citation searching. Duplicate papers were removed, and the remaining articles underwent a title-abstract screening, resulting in 67 articles that were screened in full text. Of these, 53 reports did not meet the inclusion criteria and were therefore excluded (Figure 1).

**Figure 1:**
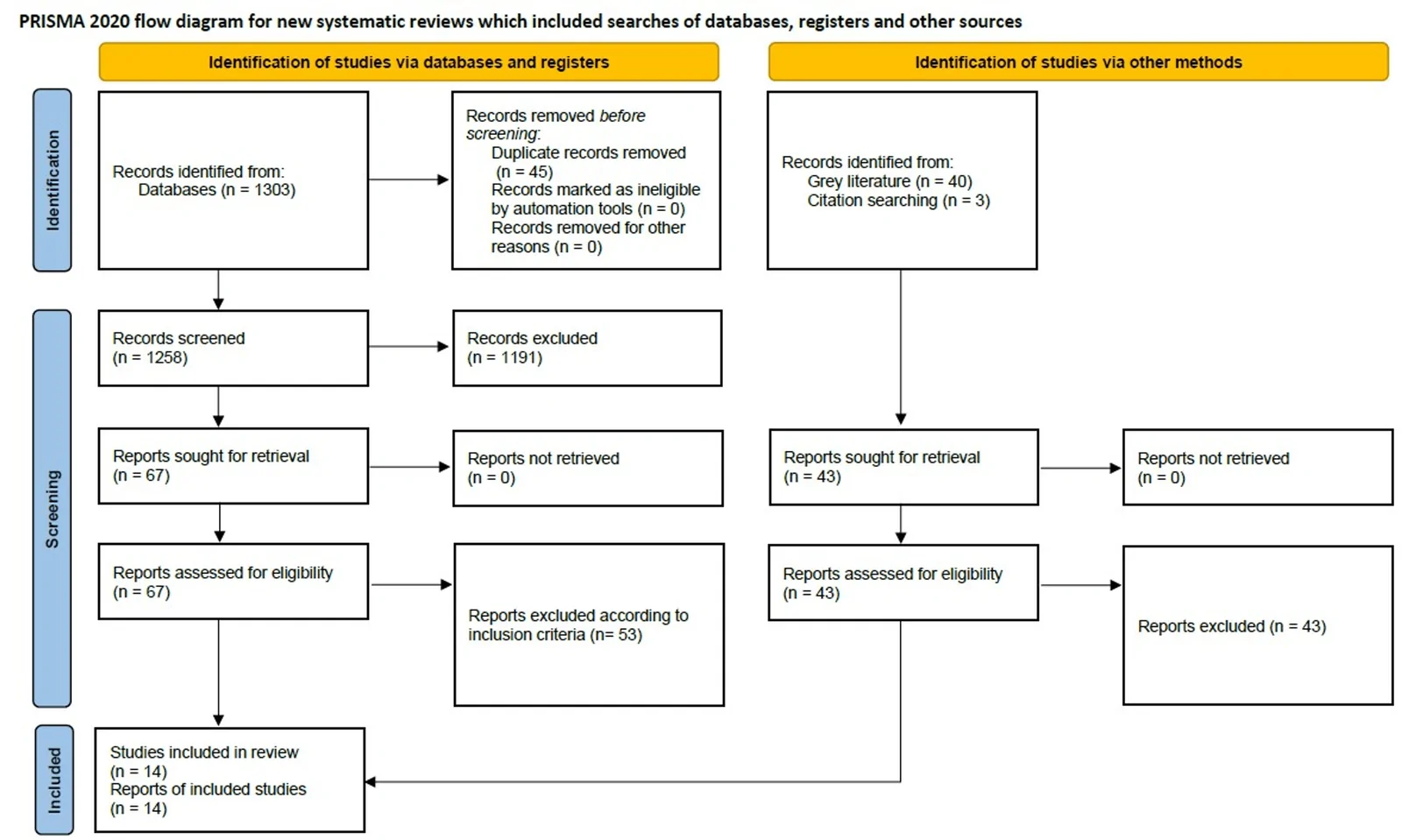
PRISMA flowchart PRISMA 2020 flow diagram of study identification, screening, and selection. The literature search of MEDLINE (via PubMed), Scopus, and grey literature (ProQuest Dissertations & Theses Global) yielded 1,346 records. After removal of duplicates, titles and abstracts were screened, and 67 articles were assessed in full text. Of these, 53 were excluded for not meeting the inclusion criteria, leaving 14 studies included in the systematic review. PRISMA: Preferred Reporting Items for Systematic Reviews and Meta-Analyses; MEDLINE: Medical Literature Analysis and Retrieval System Online

In total, 14 reports were included [4,16-28], while data from 10 studies were used for the meta-analysis [17-23,25,26,28]. Research studies employed a variety of deep learning models to evaluate and compare their effectiveness in detecting ROP with different network structures. Most studies were conducted in China, while others were conducted in Taiwan, Japan, India, Mexico, and the USA. The participants' characteristics varied widely, with studies having different criteria for including infants for screening. Two studies used the same dataset, images from the ROP Collaboration Group, which included 30 hospitals located in China [16,17], but followed different approaches to algorithms. The total number of images used varied between studies, ranging from 1,975 to 41,687. All characteristics of included studies can be found in Table 1.

**Table 1:** Included studies' characteristics ICROP: International Classification of Retinopathy of Prematurity; GA: gestational age; ETROP: Early Treatment for Retinopathy of Prematurity; BW: birth weight; ROP: retinopathy of prematurity; CNNs: convolutional neural networks; AP-ROP: aggressive posterior ROP RetCam (Natus Medical Incorporated, Pleasanton, CA, USA); Forus (Forus Health Pvt. Ltd., Bengaluru, India)

Authors - Year	Country	Algorithm	Reference standard - labeling	Patients number	Sample characteristics	Dataset	Fundus images	Test set	Fundus camera	Identification accuracy	Staging accuracy	Identification sensitivity	Identification specificity	Staging sensitivity	Staging specificity	AUC	Comparison with experts
Wang et al. - 2018 [21]	China	DeepROP (CNNs), d-Net, Gr-Net	Four ophthalmologists according to ICROP; CryoROP, ETROP	402	Low BW or premature	China	13,526	2,361	-	0.96 (clinical setting)	0.76 (minor vs severe in clinical setting)	0.96, 0.85 (clinical setting)	0.99, 0.97 (clinical setting)	0.88 (minor vs severe), 0.93 (clinical setting)	0.92 (minor vs severe), 0.73 (clinical setting)	0.99 (identification), 0.95 (staging)	The DNN model was compared to three ROP experts in identifying ROP using 2,361 images from 552 cases. The model outperformed Expert 1, achieving superior accuracy and processing each case in about 2 seconds compared to 30-60 seconds for experts.
Zhang et al. – 2019 [17]	China	VGG Google Net AlexNet	Five pediatric ophthalmologists (minimum 3 years of experience)	26,424	BW <2,000 g, BW >2000 g with severe systemic disorders	ROP Collaboration Group (China)	17,801	1,742	RetCam 2 or 3	0.97	0.84 (mild vs severe)	0.96	0.98	0.82 (mild vs severe)	0.86 (mild vs severe)	0.99 (normal-ROP), 0.92 (severe-mild)	Νot made
Hu et al. - 2019 [18]	China	Deep Neural Networks	Three ophthalmologists (minimum 3 years of experience)	720	Mean GA: 32 weeks, 32% of infants’ BW <1500 g	China	2,668	720	RetCam 3	0.98	-	0.94	0.99	-	-	0.99 (identification)	The model was compared to five pediatric ophthalmologists (2 chief physicians, 2 attending physicians, and 1 resident) and closely matched their performance. Two chief physicians showed slightly better results, particularly in precision. ROC confirmed that the model's performance was similar to three of the five ophthalmologists.
Huang et al. – 2020 [19]	Taiwan	Deep CNN, fivefold cross-validation	Three senior ophthalmologists (minimum 10 years of experience) according to ICROP	61	No ROP group: GA 28.3±2.0 weeks, Stage 1 group: GA 27.5±1.8 weeks, Stage 2 group: GA 26.0±1.8 weeks	Taiwan	1,975	244	RetCam	0.92	-	0.96	0.95	0.91 (stage 1 vs other), 0.89 (stage 2 vs other)	0.94 (stage 1 vs other), 0.98 (stage 2 vs other)	0.96 (identification), 0.93 (stage 1 vs other), 0.92 (stage 2 vs other)	Not made
Tong et al. - 2020 [26]	China	ResNet, Faster-RCNN, 10-fold cross-validation	Thirteen experts according to ICROP	-	-	China	36,231	9,772	RetCam	-	0.90 (4-degree classification), 0.95 (staging); Stage 1: 0.87, Stage 2: 0.94, Stage 3: 0.96, Stage 4: 0.99, Stage 5: 0.99	-	-	0.77 (4-degree classification)	0.93 (4-degree classification)		Comparison with two experienced retinal experts showed comparable results with one expert and better results than the other in all indices of accuracy, sensitivity, and specificity of staging.
Attallah et al. – 2021 [16]	China	DIAROP	Five pediatric ophthalmologists	890	BW< 2kg, BW >2kg with severe systemic disorders	ROP Collaboration Group (China)	17,801	-	RetCam 2 or 3	0.93	-	0.99	0.96	-	-	0.98 (identification)	Not made
Wang et al. – 2021 [28]	China	J-ROP (Deep learning model,5 classifiers)	2 trained junior ophthalmologists, one senior for disagreement resolution, and one ROP expert	8,652	GA 32.9±3.1 weeks; 4818 boys (of 7973 with known gender)	China	41,687	6,349	RetCam 2 or 3	-	-	0.94 (ROP type 1)	0.79 (ROP type 1)			0.96 (type 1 ROP) 0.91 (clinically significant ROP)	Strong correlation between expert rank ordering of overall ROP severity and i-ROP DL vascular severity score.
Huang et al. - 2021 [4]	Taiwan & Japan	VGG-19	Experts (no details mentioned) according to ICROP	210	GA< 37 weeks and/or BW ≤ 1.5 kg	Taiwan & Japan	4,714	186	RetCam	-	-			0.98 (any stage or nonstage)	0.99 (any stage or nonstage)	0.99 (staging)	The platform had an unweighted Cohen's kappa of 0.86 to 0.98, compared to 3 experienced ROP experts' Cohen's kappa of 0.93 to 0.98 on 200 retinal images extracted randomly from the test set.
Redd et al. - 2018 [24]	USA & Mexico	Deep learning image assessment system, i-ROP DL score	Ophthalmological examination by ROP experts, three experts for image labeling and consensus panel according to ICROP	870	BW 901±304 g and GA 27±2 weeks	iROP	4,861	100	RetCam	0.96	0.98 (mild vs severe)	0.96	0.95	1.0 (mild vs severe)	0.98 (mild vs severe)	-	Νot made
Zhang et al. - 2022 [25]	China	ResNet50 SE-HBP50: Deep attentive CNN	Three experienced ophthalmologists	-	-	China	12,230	3,654	RetCam 3	0.98 (ROP vs normal), 0.93 (AP-ROP vs ROP)	-	0.88 (AP-ROP vs ROP)	0.99 (ROP vs normal), 0.95 (AP-ROP vs ROP)	-	-	0.99 (identification)	Comparison with 3 ophthalmologists, in the clinical setting of 100 images. The combined model performed better than 3 experts (AP-ROP diagnostic accuracy of 100%, better than 90.00%, 85.00%, and 70.00% from the three ophthalmologists, respectively).
Peng Li et al. – 2022 [20]	China	Deep CNNs	Consensus diagnosis by three ROP experts according to ICROP	73	GA < 37 weeks and BW<2kg	China	18,827	3,680	RetCam 3	0.97	0.97	0.95	0.96	0.90 (stage 1), 0.92 (stage 2), 0.91 (stage 3)	0.97 (stage 1), 0.98 (stage 2), 0.99 (stage 3)	0.96, 0.92(stage 1), 0.98(stage 2), 0.98(stage 3)	Close to perfect agreement.
Liu et al. – 2023 [22]	China	ResNet-18 and DenseNet-121	Two senior ophthalmologists (10 years of experience) and one retinal expert for disagreements	651	Mean GA 30.9 ± 3.8 weeks, mean BW 1546.3 ± 709.5 g, 60.2% male	China	24,495	2,422	RetCam 2 or 3	0.88	-	0.85	0.91	-	-	0.95	The mean accuracy of the 4 ophthalmologists for tasks of ROP identification, severe ROP identification, and treatment modalities identification (retinal laser photocoagulation or intravitreal injections) was lower than that of the AI system.
Rao et al. - 2023 [23]	India	Convolutional neural networks (EfficientNet-B0)	Trained ROP graders according to ICROP	5,944	-	KIDROP tele-ROP screening program	25,982	7,489	RetCam 3 or Forus	0.91	-	0.91	0.91	-	-	0.97	Not made
Feng et al.- 2024 [27]	China	Semi-supervised deep learning model		473	Mean GA 29.86 ± 2.47 weeks, mean BW 1379.95 ± 412.95g, male:female 1.2:1	China	4,796	386	RetCam	-	0.78 (average) 0.72 (stage 1) 0.68 (stage 2) 0.78 (stage 3)	-	-	0.78 (average) 0.85 (stage 1) 0.58 (stage 2) 0.71 (stage 3)	0.80 (average) 0.69 (stage 1) 0.75 (stage 2) 0.79 (stage 3)	0.86 (staging -average) 0.84 (stage 1) 0.76 (stage 2) 0.84 (stage 3)	Not made

Several studies investigated the efficacy of algorithms not only for ROP identification but also for staging. Six studies focused on ROP identification [16,17,22-25], among which one identified the presence of aggressive posterior ROP [25] and another focused on ROP type 1 [24]. Studies that specifically researched ROP staging classified images as stage 1-5 according to the International Classification of Retinopathy of Prematurity (ICROP) [26], stage 1-3 [20,27], or as 'non-stage' and 'any stage' [28]. Several studies proposed stages as 'mild/minor' or 'severe/urgent' [17,18,21,24], whereas others focused on stages 1-2 [4]. The databases of the studies were not accessible, so the indices were not measured independently.

AI Performance in ROP Identification

The median accuracy across eleven studies that investigated ROP identification was 0.96. The minimum accuracy was 0.88, while the maximum was 0.98. Attalah et al. [16] created an automated deep learning-based diagnostic tool, “DIAROP,” which achieved an accuracy of 0.93 and an AUC of 0.98 for identifying ROP. The proposed system uses four pre-trained convolutional neural network (CNN) models (ResNet50, Inception, and Inception ResNet), compares their performance, and merges them by integrating spatial-spectral features that have the highest impact on improved diagnostic capability. The dataset used was ROP images collected from the ROP Collaboration Group (30 hospitals located all over China). The same dataset was used by Zhang et al. [17], who used a single CNN. Zhang et al. focused on evaluating individual CNNs, such as VGG, GoogleNet, and AlexNet, for ROP detection and achieved an ROP identification accuracy of 0.98. Redd et al. [24] aimed at specifically identifying ROP type 1 but did not report accuracy metrics. This was the only study to focus on type 1 ROP, using deep learning techniques to differentiate between type 1, type 2, and “clinically significant” ROP, a composite of type 1 and type 2 ROP and pre-plus disease. The system was trained on posterior pole vascular morphology to detect plus disease, having a retinal vascular abnormality score called the “i-ROP plus score”. Given that this algorithm accomplishes high accuracy metrics without being trained to identify a zone or stage, it was assumed that severe ROP correlates with alterations in the posterior vasculature. When compared with human experts, the i-ROP Deep Learning vascular severity score is strongly correlated with an expert ranking of overall disease severity in a dataset of 100 images.

The only study that focused on AP-ROP diagnosis, as well as overall ROP identification, was conducted by Zhang et al. [25]. An automated AP-ROP diagnosis system was proposed, which uses two independent networks; network 1 is responsible for identifying whether there is ROP in fundus images, and network 2 is classifying ROP fundus images into AP-ROP and regular ROP. The proposed system achieved 0.98 accuracy when identifying ROP and 0.93 accuracy during development for identifying AP-ROP. When applied in a clinical environment of 100 images, the diagnostic accuracy of the system increased to 100% and outperformed 3 experienced ophthalmologists.

Huang et al. [4] achieved one of the highest diagnostic metrics for the identification and staging of ROP. They used a VGG19 model after applying transfer learning to five deep neural network architectures (VGG16, VGG19, MobileNet, InceptionV3, and DenseNet) for identifying ROP with 96.0% accuracy. The same study also classified the severity of the disease with high metrics, showing 98.82% accuracy. However, there was no comparison with retinal experts.

The highest diagnostic accuracy in an algorithm was reported by Zhang et al. [17], who recorded 0.988 accuracy. A dataset consisting of 17,801 fundus images was used to train and test a deep learning algorithm with a transfer learning scheme of three classifiers (AlexNet, VGG-16, and GoogleNet). After comparing the results with five retinal specialists, VGG-16 showed the best performance. According to their ROC curves, the final model reached 0.93 precision, and its metrics were comparable to those of three out of five retina specialists. Distorted retinal images due to marginal vagueness and light exposure or optical artifacts lead to misclassifications, especially in early-stage ROP images.

Liu et al. [22] analyzed over 24 thousand images and developed a system (ResNet-18 and DenseNet-121) for diagnosing and recommending treatment for ROP. The system achieved high accuracy in ROP identification (AUC 0.9531), severe ROP identification (AUC 0.9132), and recommending treatment modalities (AUC 0.9360). Specifically, the AI system achieved significantly higher accuracy than ophthalmologists in diagnosing mild ROP and treatment-requiring ROP, except for groups with no ROP. The authors compared the performance of their AI system with four experienced ophthalmologists using an additional set of 200 RetCam images (Natus Medical Incorporated, Pleasanton, CA, USA). The AI system outperformed the ophthalmologists of different experience levels in all three tasks (ROP identification, severe ROP identification, and treatment modalities identification). Ophthalmologists had accuracies of 56%, 65%, 71%, and 76%, respectively, while the AI system scored 92% in all three tasks.

Rao et al. [23] developed a deep learning algorithm (CNN, EfficientNet-B0) for ROP detection with a high sensitivity of 0.9146 using a tele-ROP screening dataset. The algorithm performed effectively with images of the temporal fundus, a choice based on anatomical susceptibility and imaging efficiency.

AI Performance in ROP Staging

Eight studies staged ROP images according to severity with different staging methods [4,18-21,26-28]. The median accuracy across eight studies was 90%, while the median sensitivity was 89%. The staging of ROP varied greatly between studies, including differing classifications of “mild/minor” and “severe/urgent” ROP or specific stages according to ICROP as well. Staging identification varied across studies with different definitions of mild and severe disease. Specific stage identification for all stages was only made by one study [26], while other studies investigated stages 1-2 [5] or 1-3 [20,27]. Tong et al. [26] also proposed a four-degree classification (normal, mild, semi-urgent, and urgent).

Wang et al. [28] investigated multiple parameters of ROP images from four centers in China, including the stage of ROP, presence of preretinal hemorrhage, pre-plus/plus disease, posterior disease, and image quality, with a multidimensional, deep learning platform consisting of five independent classifiers, which all performed well. Referral-warranted ROP was also included, which was automatically formed by the combination of the data of the classifiers. Specifically for ROP staging, an AUC of 99% was achieved. However, staging was vaguely described as “any stage or non-stage," and no specific stage classification was performed. The performance of the platform, JSIEC Platform for Retinopathy of Prematurity (J-PROP), was further validated by comparing it to that of qualified ROP professionals, with Cohen's unweighted kappa values of 0.86 to 0.98, compared to the ROP experts' Cohen's kappa of 0.93 to 0.98. Weak-contrast lesions and artifacts were noted to interfere with the assessment of ROP staging and hemorrhage detection; however, J-PROP was unaffected by artifacts.

Huang et al. [4] focused on ROP stage 1 and 2 differentiation and reported a system that achieved an accuracy of 92%. Inadequate visualization of the stage due to the field of vision, haziness in the periphery, optical artifacts, or excessive light exposure were reported as indicators of misclassification cases.

Tong et al. [26] used two CNN models, 101-layer ResNet and Faster-RCNN, for staging and identification of ROP, respectively. ROP grading was described as follows: normal, mild (stage 1 or 2, no plus disease; routine observation); semi-urgent (stage 1 or 2, with plus disease; suggested referral); and urgent (stage 3 or 4 or 5, with or without plus disease; urgent referral for treatment). The system achieved an accuracy of 0.883, 0.900, 0.957, and 0.870, respectively, for each of the aforementioned stages of ROP severity classification. For specific stage classification (stage 1 to stage 5), the accuracies were 0.876, 0.942, 0.968, 0.998, and 0.999, respectively. The system’s performance was equivalent to or better than that of two experienced retinal specialists, showing comparable results with one expert and better results than the other in all indices of accuracy, sensitivity, and specificity of staging. Misclassification cases were significantly fewer with the intelligent system than with human experts. In urgent cases, the intelligent system had a higher accuracy than the two retinal experts (0.956 vs. 0.923 and 0.920, respectively), while in normal cases, human experts were more accurate than the system (0.929 and 0.914 vs. 0.870).

Hu et al. [18] defined mild ROP as stage 1 or 2 and severe ROP as stage 3-5, like Huang et al. [4]. Recognition of the ROP presence resulted in higher accuracy (97%), while staging ROP was satisfactory but not as good, presenting an accuracy of 84%. Wang et al. [21] applied their algorithm in a telemedicine-based clinical setting with results showing better accuracy in identifying ROP (95.6%) rather than staging (76.4%). They identified “minor ROP” as zone 2 or 3 and stage 1 or 2, and “severe ROP” as threshold disease, type I, type II, or AP-ROP, and stage 4 or 5. 

Staging ROP using deep learning algorithms was also proposed by Li et al. [20], who utilized quantitative analysis of extracted features, resulting in a high accuracy of diagnosis for ROP stages 1 to 3. Features like the width of the demarcation line or ridge, as well as the ratio of vascular proliferation, were extracted and evaluated. The more advanced the stage, the higher the diagnostic metrics of sensitivity and specificity were. When evaluating the system's impact, it was noted that incorporating its predictions into clinical decision-making showed an improvement in Cohen's kappa coefficient, reflecting enhanced consistency in diagnostic decisions. However, this research used only high-quality images of preterm infants.

The only study presenting a semi-supervised deep learning model for staging ROP was conducted by Feng et al. [27]. Experimental results showed that utilizing both labeled and unlabeled data, the model achieved near fully supervised performance. Prediction consistency and semantic structure consistency increased the model’s classification accuracy across ROP stages.

Meta-Analysis

To conduct the meta-analysis, summary characteristics were extracted from the included studies, which can be found in Table 2, containing all the relevant data of 10 studies [17-23,25,26,28]. We performed the meta-analysis only on ROP identification, as the retrieved data allowed.

**Table 2:** Meta-analysis data

First author- Year	Number of images	True positive (TP)	False positive (FP)	True negative (TN)	False negative (FN)	Sensitivity = TP/ (TP+FN)	Specificity = TN/ (TN+FP)	Overall accuracy	
Wang et al. – 2018 [21]	298	144	1	148	5	96.64%	99.33%		96.00%
Hu et al. – 2019 [18]	406	195	4	199	8	96.00%	98.00%		97.00%
Zhang et al. – 2019 [17]	1,742	158	11	1,563	10	94.10%	99.30%		98.80%
Huang et al. – 2020 [19]	101	57	2	40	2	96.60%	95.20%		96.00%
Tong et al. – 2020 [26]	9,772	1,432	539	7,392	409	77.80%	93.20%		90.30%
Wang et al. – 2021 [28]	7,492	1,026	98	6,349	19	98.20%	98.50%		98.43%
Zhang et al. – 2022 [25]	3,654	1,673	14	1,940	27	98.42%	99.28%		98.88%
Peng Li. et al. – 2022 [20]	3,680	755	104	2,789	32	95.93%	96.41%		97.98%
Liu et al. – 2023 [22]	2,422	1,129	92	1,016	185	85.92%	91.67%		88.55%
Rao et al. – 2023 [23]	7,489	1,998	466	1,839	187	91.46%	91.22%		91.29%

The data synthesis included 37,057 images, of which 9,451 were identified as positive for ROP. The prevalence of the disease in the data from these ten studies was equal to 0.26.

The heterogeneity index I² was moderate, equal to 0.75. The pooled estimate of sensitivity was 95% (95% confidence interval (CI) 91.5% - 97.1%), and the pooled estimate of specificity was 97.4% (95% CI 95.3% - 98.6%). The LR+ was over 718 (95% CI 238.4 - 2223.3), indicating a large and conclusive probability, as values above 10 are considered large for LR+. Similarly, the LR- was almost equal to 0.05 (95% CI 0.029 - 0.09), indicating a very small probability of a mistakenly classified positive case. Finally, FPR values were estimated to be equal to 0.026 (95% CI 0.014 - 0.047). HSROC pooled estimates showed similar estimates across all indices.

Figures 2, 3 represent the respective forest plots for the pooled estimates of sensitivity and specificity. All studies indicate statistically significant findings, and the left margin sensitivity scores range from 0.76 to 0.97, while the upper margin ranges from 0.80 to 1.00. The extracted data indicates consistently high sensitivity values, all at least 0.76, and high specificity values, all no less than 0.84. Regarding specificity, all studies indicate statistically significant findings, and the left-margin specificity scores range from 0.84 to 0.99, while the upper margins range from 0.92 to 1.00.

**Figure 2:**
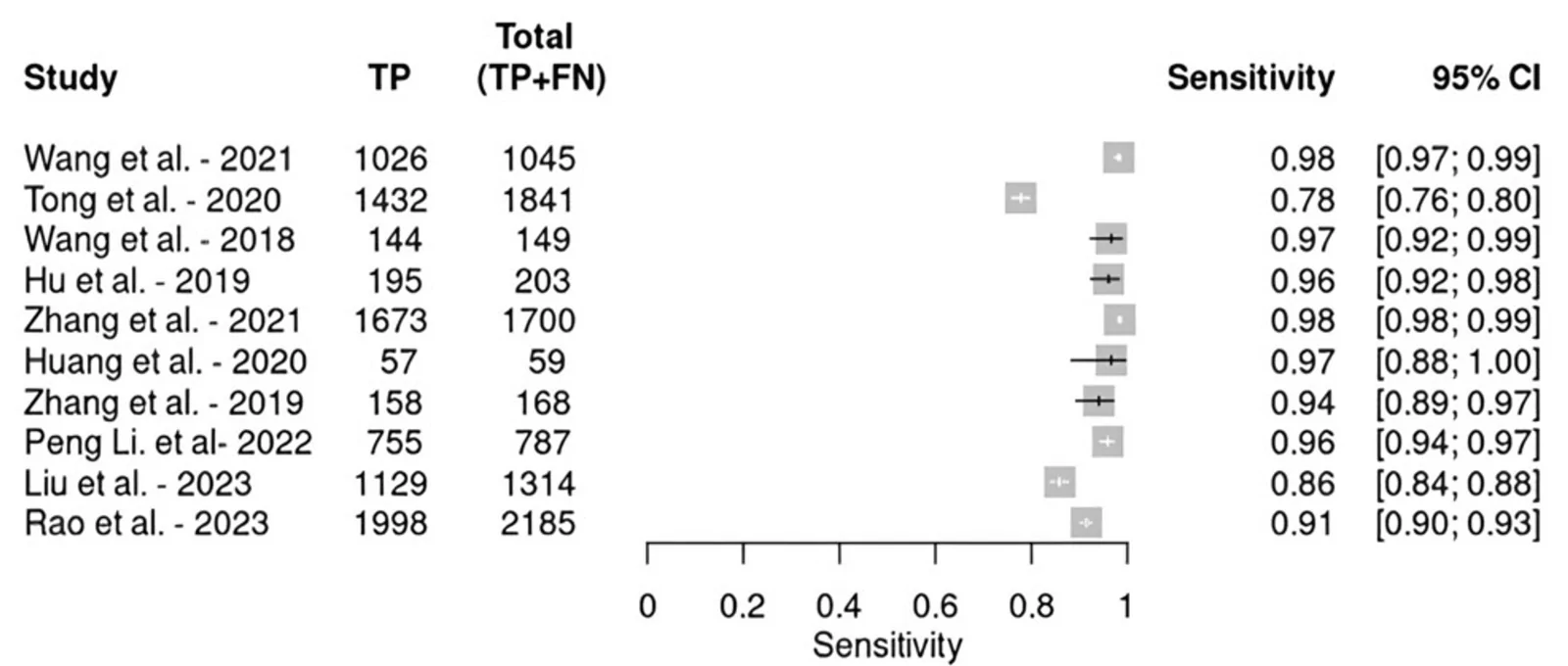
Forest plot (sensitivity) Forest plot of sensitivity for the identification of retinopathy of prematurity (ROP) by artificial intelligence algorithms across the 10 studies included in the meta-analysis. Squares represent the point estimate of sensitivity for each study with corresponding 95% confidence intervals (horizontal lines); the pooled estimate is shown at the bottom. Individual study sensitivities ranged from 0.76 to 0.97, with all estimates statistically significant, yielding a pooled sensitivity of 95.0% (95% CI 91.5–97.1%). Studies included: [17-23,25,26,28]. TP: true positive, FN: false negative

**Figure 3:**
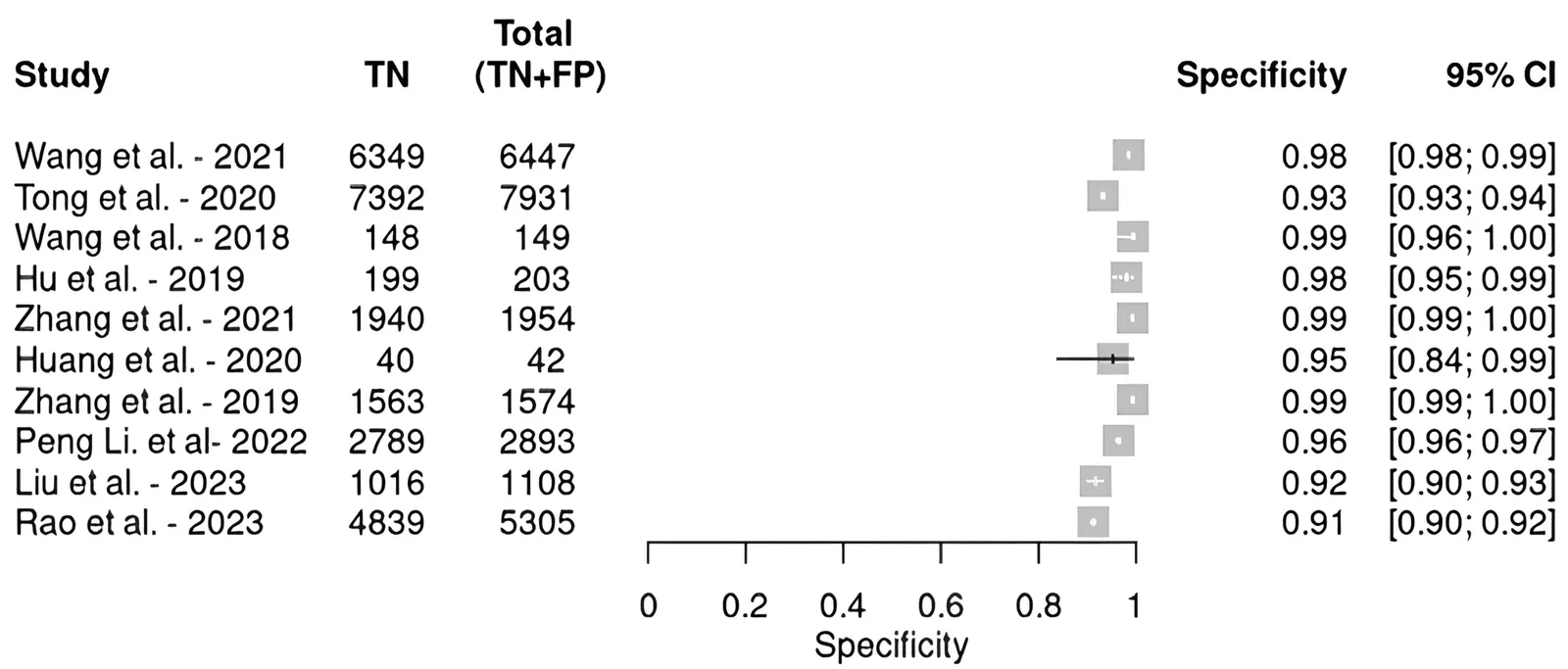
Forest plot (specificity) Forest plot of specificity for the identification of ROP by artificial intelligence algorithms across the 10 studies included in the meta-analysis. Squares represent the point estimate of specificity for each study with corresponding 95% confidence intervals (horizontal lines); the pooled estimate is shown at the bottom. Individual study specificities ranged from 0.84 to 0.99, with all estimates statistically significant, yielding a pooled specificity of 97.4% (95% CI 95.3–98.6%). Studies included: [17-23,25,26,28]. TN: true negative, FP: false positive

The SROC curve (Figure 4b) shows the pooled estimate from the studies along with its 95% confidence ellipse. The ellipse is relatively small in the upper left corner of the graph, indicating that the results suggest high diagnostic accuracy. It is noted that the curve does not expand across the possible range since the available data are restricted to the upper left corner as well. The same applies to the HSROC curve (Figure 4a).

**Figure 4:**
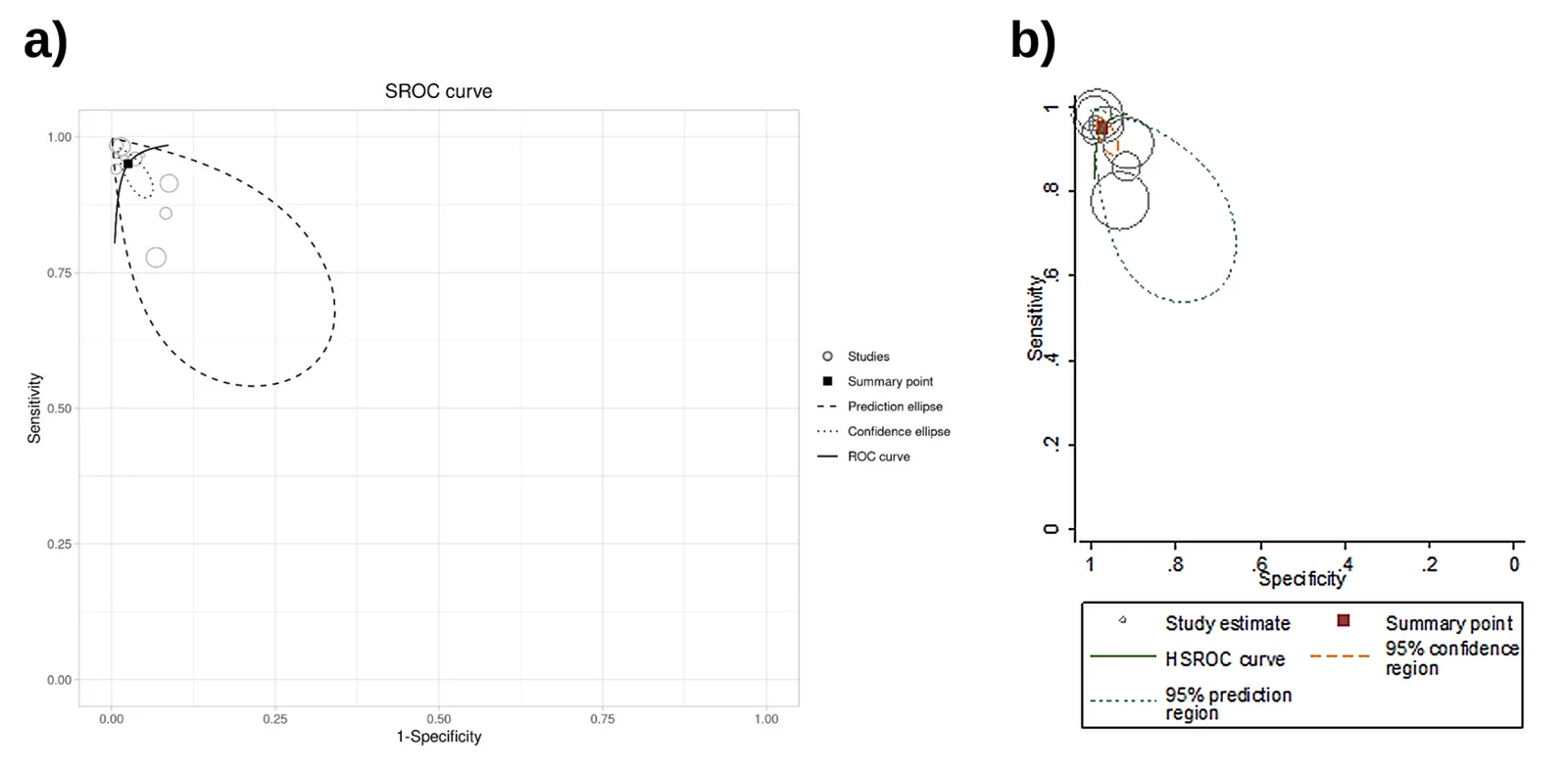
SROC and HSROC curve Summary receiver operating characteristic plots for the diagnostic accuracy of artificial intelligence (AI) algorithms in the identification of retinopathy of prematurity (ROP), based on 10 studies comprising 37,057 fundus images. (a) Bivariate summary receiver operating characteristic (SROC) curve generated in Meta-DiSc 2.0. Open circles represent the sensitivity and specificity of individual studies (circle size proportional to study sample size), and the filled square denotes the summary operating point, corresponding to a pooled sensitivity of 95.0% (95% CI 91.5–97.1%) and pooled specificity of 97.4% (95% CI 95.3–98.6%). The solid line is the fitted SROC curve, the dotted inner ellipse the 95% confidence region around the summary point, and the dashed outer ellipse the 95% prediction region; the x-axis is expressed as 1 − specificity. (b) Hierarchical SROC (HSROC) curve for the same data, generated in Stata (metandi/midas module). Circles represent individual study estimates, with circle size scaled to study weight, and the filled square denotes the summary operating point. The solid line is the HSROC curve, the short-dashed line the 95% confidence region, and the finely dotted line the 95% prediction region; here the x-axis is expressed as specificity (scaled from 1 to 0). In both panels, the clustering of study estimates and the summary point in the upper-left corner reflect the consistently high sensitivity and specificity of AI for ROP detection; because the observed data are confined to this region, neither curve extends across the full range of the plot. Studies included: [17-23,25,26,28].

The Deeks’ test that was applied regarding publication bias indicates no concerns about the effect of small-sized studies, as the t-value of 0.039 corresponds to a p-value of 0.70. The funnel plot shows rather small values of the diagnostic odds ratio (Figure 5).

**Figure 5:**
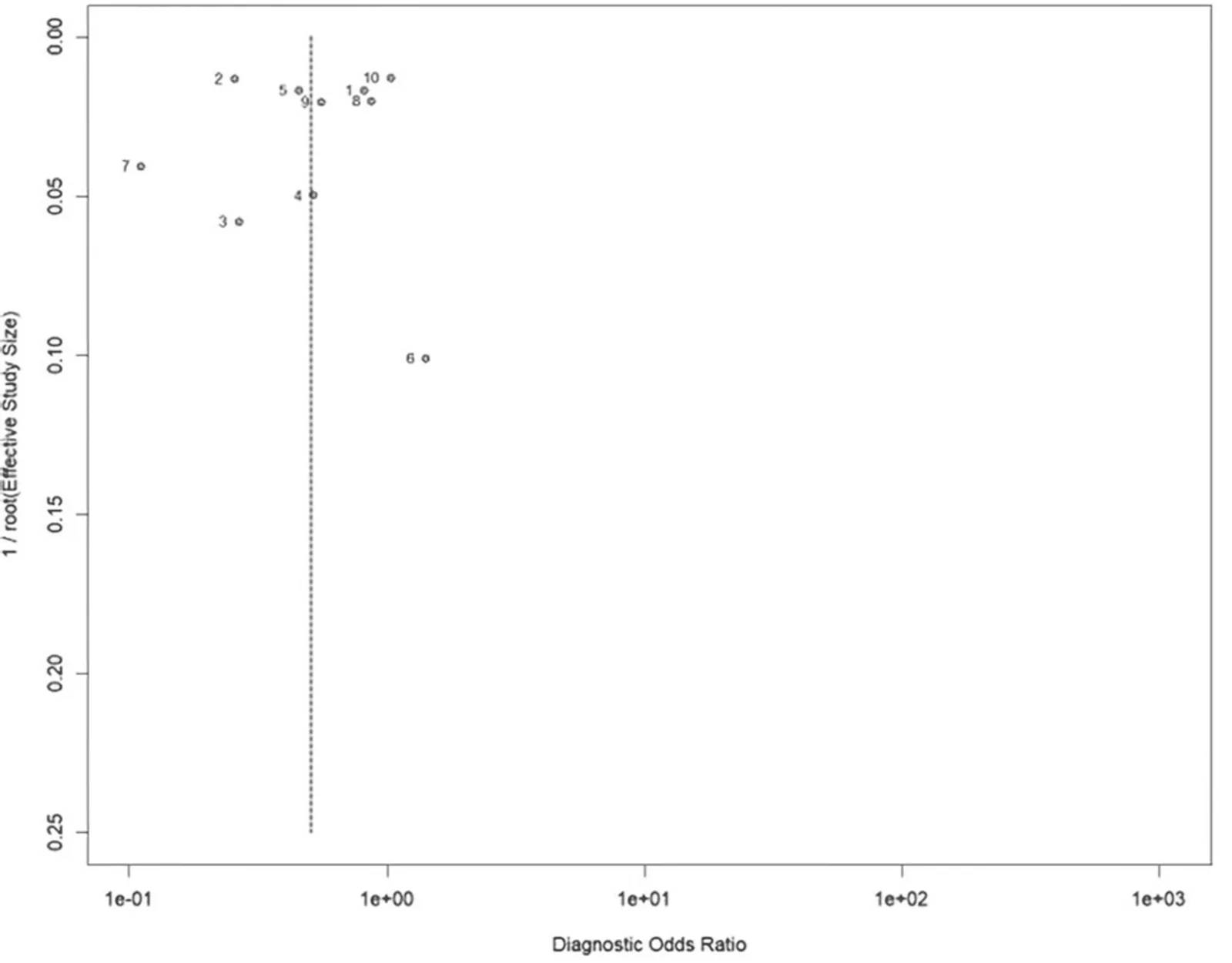
Funnel plot Deeks' funnel plot for the assessment of publication (small-study) bias across the ten studies included in the meta-analysis of ROP identification. Each numbered point represents one study; the diagnostic odds ratio (DOR, plotted on a logarithmic scale) is shown against 1/√(effective study size, ESS), with the solid line representing Deeks' regression line. The approximately symmetric distribution of points and the non-significant regression test (t = 0.039, p = 0.70) indicate no evidence of small-study effects or publication bias. Studies included: [17-23,25,26,28]. DOR: diagnostic odds ratio; ESS: effective study size

Risk of Bias Assessment

Table 3 presents a ROB assessment of various studies using the PROBAST tool, evaluating them across domains such as participants, predictors, outcomes, and analysis, as well as their applicability to clinical practice.

**Table 3:** PROBAST results PROBAST: Prediction Model Risk of Bias Assessment Tool; ROB: risk of bias. + indicates low ROB/low concern; − indicates high ROB/high concern; ? indicates unclear ROB/unclear concern

Study	ROB				Applicability			Overall	
	Participants	Predictors	Outcomes	Analysis	Participants	Predictor	Outcome	ROB	Applicability
Attallah et al. - 2021 [16]	+	+	?	?	-	+	-	?	-
Wang et al. - 2021 [28]	+	+	?	-	-	+	?	-	-
Huang et al.- 2021 [4]	+	+	+	?	-	+	-	?	-
Tong et al. - 2020 [26]	-	+	?	?	-	+	?	-	-
Redd et al. - 2018 [24]	+	?	+	-	-	+	?	-	-
Wang et al. - 2018 [21]	+	+	?	?	+	+	+	?	+
Hu et al. - 2019 [18]	+	+	?	+	-	+	-	?	-
Zhang et al. - 2022 [25]	?	+	?	?	?	+	-	?	-
Huang et al. - 2020 [19]	+	+	+	?	-	+	?	?	-
Zhang et al. - 2019 [17]	+	+	+	+	-	+	+	+	-
Peng Li. et al. - 2022 [20]	+	+	+	+	-	+	+	+	-
Feng et al. 2024 [27]	+	+	+	?	?	+	?	?	?
Liu et al. - 2023 [22]	+	+	+	?	-	+	?	?	-
Rao et al. - 2023 [23]	?	+	+	+	?	+	?	?	?

Studies like Wang et al. [21] and Li et al. [20] demonstrated low ROB across all domains, indicating strong methodological rigor and clinical relevance, while Zhang et al. [17] showed low bias with minor concerns in outcomes. In contrast, other studies [16,18,19,22-26,28] exhibited high or unclear ROB in multiple domains, particularly in outcomes and analysis, highlighting potential limitations in study design or reporting. Common issues included unclear or high-risk assessments for outcomes and analysis, indicating limitations in how outcomes were defined, measured, or analyzed. Although the Participants and Predictors domains generally showed low ROB, several studies had concerns about applicability, particularly regarding outcomes, suggesting their findings might not be broadly applicable to different clinical contexts. Notably, none of the studies reported compliance with SPIRIT-AI or CONSORT-AI guidelines [29], which are designed to address biases in AI systems and could be crucial for determining the safety and efficacy of AI interventions.

All the images were of newborns and were acquired with RetCam in all studies, ensuring consistent data quality and minimizing selection bias. Furthermore, since the images were captured from multiple sections of the fundus and by different operators, there is a potential for variability in image quality, which could influence the accuracy of ROP grade classification. While the images themselves are objective, differences in quality may impact the staging of ROP.

Clinical applicability and generalizability are also crucial when it comes to AI-ROB, which was only tested by one study [21]. Also, external data validation was poorly reported in most studies, and some studies also lacked algorithm comparison against professionals, hindering generalizability. Therefore, the majority of studies were characterized as “high concern” for their applicability.

Authors only stated that the patients’ parents/guardians consented to participation, but none of the studies specify whether their datasets were collected following gender or race bias control. In their report, Hu et al. [18] mention that only the tests where all ophthalmologists have provided consistent labels were utilized to train the algorithm to minimize bias. However, the other studies did not address potential database bias, which is crucial for real-world application and equitable access to accurate results.

Discussion

This review and meta-analysis of AI algorithms’ performance in the detection and grading of ROP shows promising results. AI demonstrates great potential in assisting with timely ROP diagnosis and staging, showing high accuracy rates for identification and staging across several studies. Specifically, the FPR in our analysis was estimated to be 0.026 (95% CI: 0.014 - 0.047), indicating a rather small probability of a false alarm. The data extracted further indicate consistently high sensitivity values, all at least 0.76, and high specificity values, all no less than 0.84, underscoring the strong diagnostic capabilities of AI systems in ROP detection. These performance metrics emphasize the potential of AI to improve diagnostic accuracy and reduce the burden of missed or misdiagnosed cases. Nonetheless, staging of ROP showed significant heterogeneity among studies, highlighting the need for a standardized method for comparison.

At this point, ROP frequency is rising, and resources for timely diagnosis are becoming scarcer [30], so there is a growing need for a fast, efficient diagnostic tool to assist in ROP detection. Due to the substantial intra- and inter-expert variability in interpreting images [31], systems that can provide objective assistance in diagnosis can be highly valuable. It is questionable if currently available algorithms can lessen the level of uncertainty inherent in diagnoses. Additionally, the question arises as to whether these algorithms can practically support clinicians, particularly in regions with limited resources and high disease prevalence, where access to specialized care and screening remains constrained.

Our findings are consistent with the previous systematic reviews [32,33], which also reported high diagnostic accuracy of AI for ROP detection. However, our review incorporates more recent studies and therefore provides updated pooled estimates that reflect the latest developments in the field. In addition, our meta-analysis focuses specifically on ROP identification, rather than combining multiple ROP subtypes, offering a clearer and more homogeneous evaluation of diagnostic performance.

Several limitations should be considered when interpreting our findings. First, the original datasets were not accessible, and the analysis therefore relied on diagnostic performance measures reported in the individual studies. Incomplete reporting of diagnostic metrics also prevented the inclusion of all eligible studies in the meta-analysis. Furthermore, methodological heterogeneity was observed across studies, including differences in sample size, unit of analysis, algorithm architecture, outcome definitions, and ROP severity classification. To reduce heterogeneity, the meta-analysis was restricted to ROP identification and excluded studies reporting only plus disease or vascular severity scores. For studies evaluating multiple classifiers, the optimal reported performance estimates were included. Consequently, quantitative synthesis was possible for 10 studies reporting sufficient data on ROP identification.

It is also important to note that comparison with experts was made against individual experts, not necessarily a consensus among them. This raises an important consideration: while the AI system shows potential, there are borderline cases in treatment-requiring ROP according to Early Treatment for Retinopathy of Prematurity (ETROP) criteria where even expert ophthalmologists may disagree. In practice, the final decision for treatment lies with the attending physician, which introduces a level of subjectivity not fully captured in the AI comparison. This distinction is crucial in interpreting the results of the study, as it affects how we view the AI's real-world applicability.

The studies included in the analysis were highly susceptible to bias due to the lack of application of their algorithms in real-world clinical settings. Studies may be prone to bias when algorithms are only tested in controlled environments, as these conditions fail to capture the complexity and variability of real-world clinical settings. Without real-world validation, the algorithm's performance may be overestimated and its applicability limited.

Incorporating AI in clinical practice requires careful consideration of ethical dimensions such as fairness, transparency, generalizability, and bias controls, especially in low- and middle-income countries where many individuals with vision loss reside. Addressing these ethical implications is critical, as disparities in eye health are shaped by a complex interplay of factors including gender norms, ethnicity, socioeconomic status, and insufficient bias controls. Before widespread implementation, it's crucial to establish strong oversight and regulatory frameworks that can adjust to the new landscape of an AI-integrated clinical environment. Although AI has effectively detected ROP, demonstrating high indices of performance as shown in the studies collected and analyzed, there is still research needed regarding the applicability and bias of these algorithms.

Furthermore, we recognize that while many algorithms included in our meta-analysis demonstrate strong diagnostic accuracy for ROP identification, they do not necessarily fulfill the strict criteria to be considered screening tools according to the World Health Organization’s criteria for screening [34]. In the context of ROP, validated intervention points include threshold ROP, Type 1 ETROP, and AP-ROP as defined by ICROP1 and ICROP2. The algorithms analyzed in our study do not explicitly target these specific intervention thresholds but rather focus on feature detection or general disease severity scoring. Hence, while these algorithms are diagnostically valuable, they may serve more accurately as diagnostic support tools or triage tools, rather than as formal screening tests. Evaluation of AI models specifically trained and validated against these clinical inflection points should evaluate their use as screening tools.

This systematic review and meta-analysis highlight the early, albeit promising, efforts to integrate AI into ophthalmology. It underscores the need for global coordination and agreement on a standardized staging system to ensure that results are generalizable across different users. Given the urgent need for AI assistance in ROP evaluation, there is a clear necessity for better-structured studies to minimize bias and enhance the utility of their findings. Additionally, the review reveals a significant gap in the practical application of this technology in clinical settings, which presents its own set of challenges.

Large-scale trials of proposed systems have yet to be conducted in a clinical setting, and thus, the reproducibility of results remains limited. In summary, AI shows great potential in assisting with timely ROP diagnosis and staging. Yet, real-world implementation faces challenges, including ethical and economic factors. While AI is expected to enhance medical quality and save costs in ophthalmology, its adoption remains low despite increasing research due to limited economic evaluations that can make policymakers reluctant to adopt new healthcare technologies. Therefore, evaluation studies should also be conducted, following the large-scale, externally validated studies needed to prove AI applicability and efficacy.

## Conclusions

AI has revolutionized the field of ophthalmology by harnessing the power of computational algorithms for disease detection, especially for ROP, by providing a powerful tool for early detection. Our systematic review and meta-analysis provide a thorough view of the current literature on ROP diagnosis with AI, as well as its limitations and potential. AI facilitates precise and efficient diagnosis and enhances staging programs, potentially revolutionizing healthcare delivery. Integrating AI into medical practices benefits both patients and ophthalmologists, improving healthcare access and providing reliable tools for ophthalmology clinics. The intersection of AI with ophthalmology represents a promising avenue for improving patient care and clinical outcomes but requires attention and careful oversight to implement in real-world settings.

## Appendices

Appendix 1

The search string used in PubMed (MEDLINE) was the following:

((((((((("retinopathy of prematurity"[MeSH Terms]) OR ("ROP"[All Fields])) OR ("retinopathy of prematurity"[All Fields])) OR ("retinopathy of prematurity diagnosis"[All Fields])) OR ("retinopathy of prematurity diagnostic imaging"[All Fields])) OR ("rop stage"[All Fields])) OR ("rop zone"[All Fields])) OR ("rop vascular severity"[All Fields])) OR ("rop vascular severity score"[All Fields])) OR ("rop type"[All Fields]) AND ((((((("artificial intelligence"[MeSH Terms]) OR ("algorithm*"[All Fields])) OR ("machine learning"[All Fields])) OR ("deep learning"[All Fields])) OR ("support vector machines"[All Fields])) OR ("convolutional neural network*"[All Fields])) OR ("classifier*"[All Fields])) OR ("automated neural network*"[All Fields])
